# Semantic Priming in Mild Cognitive Impairment and Healthy Subjects: Effect of Different Time of Presentation of Word-Pairs

**DOI:** 10.3390/jpm10030057

**Published:** 2020-06-29

**Authors:** Valeria Guglielmi, Davide Quaranta, Ilaria Mega, Emanuele Maria Costantini, Claudia Carrarini, Alice Innocenti, Camillo Marra

**Affiliations:** 1 Neurology Unit, Fondazione Policlinico Agostino Gemelli-IRCCS, 00168 Rome, Italy; valeria.guglielmi@policlinicogemelli.it; 2 Department of Neuroscience, Catholic University of Sacred Heart, 00168 Rome, Italy; ilaria.mega@gmail.com (I.M.); emanule.m.costantini@gmail.com (E.M.C.); claudia.carrarini@live.it (C.C.); linceinnocenti@gmail.com (A.I.); camillo.marra@policlinicogemelli.it (C.M.); 3 Memory Clinic, Fondazione Policlinico Universitario Agostino Gemelli-IRCCS, 00168 Rome, Italy

**Keywords:** Alzheimer disease, semantic priming, mild cognitive impairment

## Abstract

**Introduction**: Semantic memory is impaired in mild cognitive impairment (MCI). Two main hypotheses about this finding are debated and refer to the degradation of stored knowledge versus the impairment of semantic access mechanisms. The aim of our study is to evaluate semantic impairment in MCI versus healthy subjects (HS) by an experiment evaluating semantic priming. **Methods**: We enrolled 27 MCI and 20 HS. MCI group were divided, according to follow up, into converters-MCI and non converters-MCI. The semantic task consisted of 108 pairs of words, 54 of which were semantically associated. Stimuli were presented 250 or 900 ms later the appearance of the target in a randomized manner. Data were analyzed using factorial ANOVA. **Results**: Both HS and MCI answered more quickly for word than for non-word at both stimulus onset asynchrony (SOA) intervals. At 250 ms, both MCI and HS experienced a shorter time of response for related-word than for unrelated words (priming effect), while only the converters-MCI subgroup lost the priming effect. Further, we observed a rather larger Cohen’s d effect size in non converters-MCI than in converters-MCI. **Conclusion**: Our data, and in particular the absence of a semantic priming effect in converters-MCI, could reflect the impairment of semantic knowledge rather than the accessibility of semantic stores in MCI individuals that progress to dementia.

## 1. Introduction

Alzheimer’s disease (AD) is a degenerative process that, in its typical evolution, proceeds in the early stages from the medial perirhinal cortex to the entorhinal, and finally, the hippocampus cortex [1]. Memory disorders are consequently the first symptoms of the disease and episodic memory has been always considered a neuropsychological diagnostic marker of AD. Since the medial perirhinal cortex is deeply involved in the organization of item recollection and general knowledge, it is conceivable that impairment of semantic memory could precede that of episodic memory in the early stages of AD [2]. As a matter of fact, subjects with mild AD experienced lower scores on tests of object naming [3,4,5], and categorical verbal fluency [2,6,7,8]. Moreover, low performances in different semantic memory tasks have been also reported in mild AD [9,10,11,12], with categorical verbal fluency tasks being found impaired in mild cognitive impairment (MCI) [13,14]. Some authors focused on specific deficits of semantic memory emphasizing the presence of an early and specific deficit in naming and knowing of famous people in MCI patients [15]. Other authors explored specific linguistic markers of degradation of the semantic system, such as frequency, age of acquisition (AoA), familiarity and typicality of the words produced in both category fluency tasks [16,17,18] or procedural speaking in famous writers [19]. Most studies have shown that AD and MCI patients tend to produce, as the disease progresses, more frequent, more typical and early acquired words [20,21,22,23,24].

Nevertheless, the nature of semantic impairment in the early stage of AD is still controversial. Two main hypotheses have been suggested. The first refers the semantic memory deficits to progressive degradation of the stored knowledge; the second suggests an inability of patients to timely recollect the semantic knowledge cause of a deficit of the mechanism that guarantees the access to the information. In this latter case, executive and phonological verbal mechanisms are hypothesized, and worse category fluency was interpreted as a deficit in access to semantic system [25].

The two explanation are not mutually exclusive and probably describe a simple progression of the degradation of the semantic system. Duong et al. studied both automatic and intentional access to the semantic system in a cohort of MCI and AD subjects. They found that the MCI group was only impaired on tasks of intentional access while the AD group was impaired on both types of tasks. This study would suggest that impaired access to the semantic system in MCI precede semantic store degradation observed in AD.

On the other hand, some authors sustain the hypothesis of early semantic degradation from the beginning [9,26,27,28,29]. The disproportionate impairment in category fluency relative to letter-based fluency—that is particularly sensitive to executive damage [5,30]—come out on the side of this.

We have previously investigated some aspects of the semantic system, regarding semantic fluency, specifically the typicality of words produced in a semantic verbal fluency task [23]. Our results showed that the MCI group produced more typical words in comparison with the healthy group, while no differences in typicality were observed within the MCI and AD group. These results were interpreted in term of a progressive disruption of semantic system organization, leading the patients to retrieve more typical items of specific categories in respect to less prototypical elements. Our groups also reported that MCI individuals who will eventually progress to dementia produce words that are less related than the ones produced by healthy controls and stable MCI in a category fluency test [31]. This evidence can be interpreted as an effect of reduced strength of conceptual links between items belonging to a given category.

In previous works, semantic priming (SP) has been used to explore the semantic system automatically; SP is an experimental condition of a lexical decision based on the fact that subjects are faster and more accurate in recognizing a target word when it follows a related word as a prime than when it follows an unrelated word [32]. When it happens in a brief interstimulus interval (250 ms or less), the priming effect is automatic and is not influenced by preparatory or intentional strategies [33]. The prime word automatically and diffusely activates the semantic network, allowing a faster recognition of the target word. Several explanations of the priming effect have been proposed. According to the classical spread-activation hypothesis, the retrieval of an item from memory requires the activation of its conceptual representation. The activation can spread to related concepts, that in turn will be more easily retrieved [34]. The compound-cue models claim that a cue to memory contains the target item and other items of the surrounding context. In this model, a relevant effect of familiarity on priming effect is predicted, since cues formed by related words will be more familiar than cues formed by unrelated words, explaining the shorter reaction times in the case of related words [34]. Finally, distributed network models have been developed. According to the so-called proximity models, priming is observed because related primes and targets are close to each other in a high-dimensional semantic space [35]. The semantic proximity models claim that concepts are represented by patterns of activity over interconnected conceptual units, and that related concepts share similar patterns of activity. The other category of distributed models, referred to as learning models [34], attributes SP to incremental learning. In fact, each presentation of a word causes an alteration of the network connections, increasing the probability of producing the same response to the same input, including semantically related targets. According to this class of models, the decay of learning can be very slow, and may cause SP also over long SOAs. The models of SP are embedded in general conceptualizations of semantic memory based on observations obtained from other typologies of investigations. In Alzheimer’s disease and MCI, previous studies suggest that spread-activation models or more complex proximity models could be satisfactory to explain the disruption of lexical-semantic system [31,36].

The previous studies on SP in AD have reported contrasting results. Some authors found less-than-normal priming (hypo-priming) in Alzheimer’s disease patients compared with controls [37,38,39], while others reported no differences on priming in AD vs. Healthy subjects [40,41] or even paradoxical increased priming effects (hyper-priming) in Alzheimer’s disease patients [42,43,44,45,46,47]. These opposed results may reflect some differences in the methods used, and also clinical heterogeneity in the samples of individuals studied. The severity of dementia, and therefore of semantic deficits, differed from one study to another, leading to different results in semantic tasks, SP included. Furthermore, the type of paradigm between the prime and the target, that maybe, superordinate, coordinate, or attribute, seems able to influence the priming effects observed. When the semantic relationship is based on the superordinate category, a normal priming effect was observed [41,48,49], while hypo-priming is more frequently reported in experiments in which the target is an attribute of the prime [48,50,51]. Finally, when the prime and the target belong to the same category (coordinate condition) [46,48,49] hypo- or hyper priming is observed.

Few studies have investigated SP in mild cognitive impairment. Duong et al. [25] reported that MCI and healthy individuals both reported SP effect. The same authors, instead, reported that the MCI group was impaired on tasks that required intentional access to the semantic system, suggesting that in early stages of AD, the involvement of the semantic system concerns access to its first.

The aim of the present study is to clarify the subtle semantic impairment in the MCI condition by studying the behavior to an SP paradigm in comparison to a population of healthy subjects (HS). First, we want to verify if a different behavior can be observed in MCI compared with HS and to understand the meaning of the observed behavior. From a theoretical point of view, if in MCI condition patients experienced an early degradation of the semantic system, consequently they do not benefit from SP. On the contrary, if there is difficult access to the semantic system, the semantic facilitation of priming allows a faster time of answer, especially at the brief, pre-attentional interstimulus interval.

## 2. Materials and Methods

We enrolled 27 individuals with amnestic MCI and 20 age- and education-matched HS. In the patient groups, all subjects underwent a clinical evaluation including medical history, physical and neurological examination, and an extensive neuropsychological evaluation and MRI scans. All the subjects of our sample were native Italian speakers and none of them had a history of traumatic head injury, alcoholism, epilepsy, stroke, nor other relevant neurologic, psychiatric, and general medical diseases. MCI was diagnosed following clinical criteria [52]; the diagnosis of Alzheimer’s Disease was based on current clinical criteria [53]. MCI subjects were evaluated after 12 and 24 months. At follow up, patients were assessed according to the clinical dementia rating scale and activity daily living scales. Dementia was diagnosed when CDR >1 and functional impairment were found. Eleven MCI progressed to overt dementia at the 24 months follow-up.

### 2.1. Neuropsychological Examination

Patients were diagnosed as amnesic MCI after administration of the Mini Mental State examination and a comprehensive battery including learning and long term memory (Rey’s Auditory Verbal Learning test- RAVLT) [54]; executive functions (Stroop’s test; [55]); visual search and attention (Multiple Features Target Cancellation, MFTC; [56]); working memory (digit span forwards and backwards; [57]); abstract reasoning (Raven’s progressive matrices, PM’47; [54]); constructional praxis (copy of figures without and with Landmarks; [54]). Verbal fluency was examined by phonological [54] and categorical verbal fluency tasks [58].

### 2.2. Lexical and Semantic Priming Procedure

**Figure 1:**
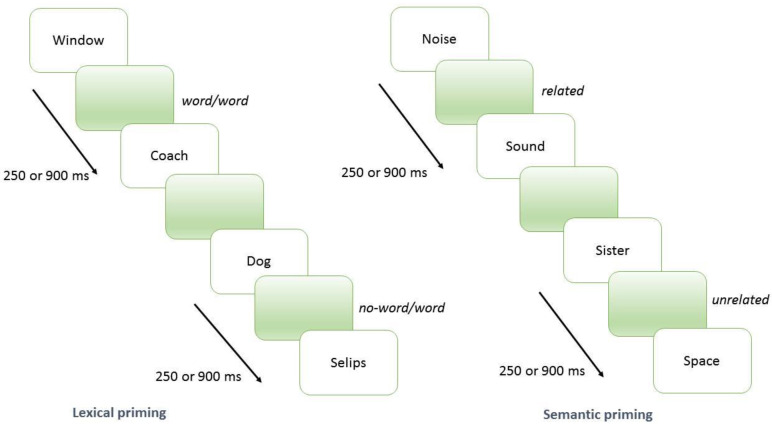
Assessment of the lexical and semantic priming tasks.

The task consisted of 216 pairs of stimuli (prime-target). Each target stimulus was a word or a non-word preceded by a prime word. Patients were required to decide if the target was a word or not as fast as possible. In total, 108 pairs were a word–non word couple and 108 pairs were a word–word couple. In total, 54 out of the 108 word–word couples were semantically related. The primer–target couples were controlled for word frequency [59]. Stimuli were presented at different intervals of response to the prime and the appearance of the target (SOA) of 250 and 900 ms (Figure 1). The order of appearance of the pairs of word–non word and word–word at different SOA was randomized for each subject. Words were presented in Italian (English translation of the words is reported in the Appendix A). Our task was built with OpenSesame software and was administered by means of a 15” laptop posed at 0.5 mt from the individual, who had to respond by pressing one out of two buttons marked with a “yes” or “no” label. Time of response for each item was recorded.

We evaluated the ‘lexicality effect’; that is, the faster recognition of words compared to non-words. The lexicality effect has been computed as the difference between the mean reaction time between response after that the priming was a word. The ‘SP effect’ was calculated as the difference in the mean reaction time to word that was preceded by semantically related or semantically unrelated word.

## 3. Results

MCI and HS were similar in age (MCI: mean age = 72.79; DS = 5.090; HS: mean age = 69.44; DS = 5.515; *p* = 0.115) and educational level (MCI: mean = 11.61; DS = 2.909; HS: mean = 13.12; DS = 2.711; *p* = 0.193). At 24 months follow up, 11 subjects (45.8%) were diagnosed as affected by Alzheimer Disease. Then, according to the progression or not to dementia, we divided MCI group in non converters-MCI (*n*. 13) and converters-MCI (*n*. 11).

### 3.1. Lexicality Effect

Both at 250 ms and 900 ms, the factorial ANOVAs comparing MCI vs. HS showed a significant effect of the GROUP factors (F = 458; *p* <0.001 and F = 441; *p*<0.001 respectively), WORD factors (word vs. non-word) (F = 1072; *p* < 0.001 and F = 1166; *p* < 0.001 respectively) and of interaction GROUP × WORD (F = 23; *p* < 0.001 and F = 42; *p* < 0.001, respectively).

Table 1 shows response times of the two groups at 250 and 900 ms. Both HS and MCI answered more quickly for word than for non-word, regardless of the presence of a semantic connection between prime and target.

Similarly, patients with MCI were significantly slower than HS both for words (*p* < 0.001) and non-words (*p* < 0.001).

In the comparison between converters-MCI, non converters-MCI and HS at 250 and 900 ms, ANOVA confirmed a significant effect of the GROUP factors (F = 230; *p* < 0.001 at 250 ms and F = 223; *p* < 0.001 at 900 ms), WORD (F = 1144; *p* < 0.001 at 250 ms, and F = 1267; *p* < 0.001 at 900 ms) and of the interaction GROUP x WORD (F = 14; *p* < 0.001 at 250 ms and F = 22; *p* < 0.001 at 900 ms). Both HS and MCI answered more quickly for word than for non-word; non converters-MCI and converters-MCI were significantly slower than HS both for words (*p* < 0.001) that for non-words (*p* < 0.001); the answers time of converters-MCI were not significantly different for the response times of subjects with converters-MCI, both in the presence of words (*p* = 0.166) and in the presence of non-words (*p* = 0.9).

#### 3.1.1. Priming Effect at SOA 250 ms

To evaluate the priming effect, we have considered only reaction times of word–word pairs, comparing semantically related to semantically unrelated pairs of words.

The ANOVA analysis (considering the whole group of MCIs) showed a significant effect of the GROUP factor (F = 131.6; *p* < 0.001) and ‘Semantic Relation’ Factor (REL) (F = 20.9; *p* < 0.001) but not of the interaction between the two (F = 0.2; *p* < 0.678). On Table 2 are reported the reaction times in the two conditions (pairs of correlated first-target or unrelated strings).

The post-hoc analysis showed that for both related pairs and unrelated words. HS answered more quickly than MCI (in both cases, *p* < 0.001). The response times for related words were lower than the response times for unrelated words in both in HS and MCI (see Table 2). The Cohen’s d show only a little effect size in both groups.

In the comparison between non converters-MCI, converters-MCI and HS, ANOVA showed a significant effect of the GROUP factors (F = 69.9; *p* < 0.001) and REL (F = 20.2; *p* < 0.001) but not of the interaction between the two (F = 0.4; *p* < 0.650). On Table 2 is reported the reaction times in the two conditions (pairs of correlated first-target or unrelated strings) in the three groups (non converters-MCI, converters-MCI and HS).

In this condition, we observed a rather larger Cohen’s d effect size in non converters-MCI than in converters-MCI. In particular, the effect size in the converters-MCI was similar to the HS group.

The post-hoc analysis showed that for both the related and the unrelated pairs of words, the HS answered more quickly than the subjects non converters-MCI or converters-MCI (*p* < 0.001 in all cases). Response times for related words were shorter than response times for unrelated words in HS and non converters-MCI, but not in converters-MCI (see Table 2). Although both non converters-MCI and HS show a similar priming effect, the Cohen’s d is different between these two groups suggesting that the priming in non converters-MCI is preserved only at the cost a higher discrepancy between the two conditions (with related and unrelated words).

#### 3.1.2. Priming Effect at 900 ms

ANOVA analysis showed a significant effect of the GROUP factors (F = 115.6; *p* < 0.001) and REL (F = 17.2; *p* < 0.001). No interaction between GROUP and REL was found (F = 0.5; *p* = 0.488). Table 2 (upper part) reports the mean values of reaction times in the two conditions in HS and MCI.

The post-hoc analysis showed that HS answered more quickly than MCI both for the related pairs and the unrelated words (*p* < 0.001). In HS, the response times for related words did not differ from response time for unrelated words, while MCI answered more quickly for related words that for unrelated words.

Comparing non converters-MCI, converters-MCI and HS, ANOVA showed a significant effect of the GROUP factors (F = 53.4; *p* < 0.001) and REL (F = 16.0; *p* < 0.001), but no interaction between the two (F = 0.96; *p* < 0.383). On Table 2 are reported the means of reaction times in the two conditions in the three groups.

The post-hoc analysis showed that HS had shorter reaction times than non converters-MCI and converters-MCI for both related and unrelated words (*p* < 0.001 in all cases).

Comparing response times for related words vs. unrelated words among the three group, non converters-MCI answered more quickly for related pairs than for unrelated ones, while HS and converters-MCI did not differ in reaction times among related and unrelated words. (see Table 2). The Cohen’s d effect size shows that this effect was higher in non converters-MCI than in bot converters-MCI and HS.

## 4. Discussion

Our data seem to confirm the hypothesis of early degradation of the semantic system in MCI.

A first consideration regards times of reaction: in all tasks, MCI answered significantly slower than HC. These findings are in line with previous studies about priming [42,43,44,49]. Despite the slower time of reactions, in our sample, lexicality effect was equally represented in HC and MCI, even after splitting MCI in non converters-MCI and converters-MCI. Lexicality effect reflects a physiological learning mechanism: the repeated exposition to verbal stimulus encourages the creation of phonological, syntactic and semantic representations of stimuli. This phenomenon does not happen when stimuli are non words. Our data confirm general findings of the integrity of the phonological, orthographic and syntactic system in early AD. Furthermore, in our study, the presence of lexicality effect in the MCI group demonstrate a good lexical activation and lexical access, even if it happens slower than in HS.

Our data show that at the shorter interval, both HS and MCI had SP effect; nevertheless, when MCI group was divided according to progression to AD, the converters-MCI group lost SP effect, while non converters-MCI had same behavior that HS. At 900 ms, HS lost priming effect, because of the intervention of attentional, strategic and inhibitory mechanisms. Non converters-MCI still showed priming effect, probably because they experienced slower reaction time, and consequently a slower activation of the attentional system. In converters-MCI no priming effect was found, as in the 250 ms interstimulus condition.

In our study MCI group globally considered does not differ from HS, partly because of the intrinsic heterogeneity of MCI population, who belong to subjects with prodromal AD and subjects without progressive memory deficit.

Nevertheless, it is very interesting that the MCI group behaved differently according to disease progression: non converters-MCI had normal priming effect as HS, while converters-MCI lost priming effect. Further, non converters-MCI experienced a larger effect size than converters-MCI; this means that non converters-MCI carry out a greater effort to maintain a SP effect in a compensatory manner. Instead, converters-MCI does not experience any compensatory strategy. This phenomenon happens both at 250 ms and at 900 ms.

These results deserve several considerations: first, the loss of priming effect in converters-MCI—that can be considered as prodromal AD—suggests that semantic system is early impaired regardless of executive functions from the beginning in AD; according to the hypothesis of a semantic store degradation, even in MCI, the priming effect disappears in our converters-MCI because the relationship within semantically close words is lost, preventing the activation of the semantic system or making it ineffective to generate priming effect. This explanation is in line with previous reports about the linguistic features of words produced in the semantic fluency task, by meaning the tendency to generate words with higher typicality and early AoA [20,21,22,23,24]. A possible explanation is that the progressive reduction of the knowledge of the attributes of objects and the relations between similar entities can cause the loss of SP effect that is the counterpart of difficulties observed in the semantic fluency tasks. In general, our findings could be accounted by spread-activation models, or proximity class of diffuse network models, in accordance to previous evidence from studies on semantic fluency [31,36]. On the other hand, learning models are not supported by our findings, since we observed a loss of SP over a long SOA in HS [34].

Some characteristics of the semantic system can help to support our findings. In their study, Mulatti et al. described the cumulative semantic effect in healthy subjects and MCI [51]. The cumulative semantic interference effect refers to a linear increase in the picture naming reaction times which is a function of the already named pictures belonging to the same semantic category to which the named picture belongs. In the author’s opinion, this phenomenon is due to the interaction between different cognitive processes involved in picture naming as shared activation, priming and competition: when a representation is activated in the lexicon upon the presentation of a picture, the lexical representations of semantically related items are also activated (shared activation); the activated no-target lexical representations compete with the target lexical representation in a mutually inhibitory way (competition), thus slowing down processing, while any retrieval of a lexical representation facilitates its subsequent retrieval (priming). Analogously, even if in a different experimental condition, in our experiment we observed lexicality effect, but not SP effect in our MCI patients that progress to AD. Our data support the evidence of a loss of cumulative semantic interference effect in MCI converters that could rely on the lack of the shared activation of the semantic features belonging to same categories of knowledge, while the lexical priming still works in the early stages of AD.

The study has several limitations, including the relatively small number of subjects included. MCI patients were diagnosed as MCI due to AD according to clinical and imaging data, but without biomarkers confirmation. However, the rather long follow-up period reduces the possibility of inaccurate identification of MCI individuals both at the baseline and at the follow-up. Furthermore, the number of MCI patients who progressed to dementia is rather low; thus, it is possible that we were not able to detect the SP effect in converters-MCI because of insufficient statistical power. Nevertheless, the number of non-converters MCI was comparable to the number of converters-MCI, yet we were able to detect a significant SP effect, with a Cohen’s d higher than the one observed in HS. However, a cautious interpretation of our results is mandatory.

## 5. Conclusions

In conclusion, our data are of same interest from a theoretical and clinical point of view. As for the theoretical significance, the SP seems a good paradigm to detect subclinical deficit of the semantic system in the early stages of the AD pathology. From a clinical point of view, the different behavior between non converters-MCI and converters-MCI at the SP suggests that this paradigm could be a practical method for evaluating semantic memory in subjects with MCI. If confirmed in larger samples, these results may have significant prognostic and therapeutic implications.

## Figures and Tables

**Table 1:** Lexicality effect.

	HS		MCI			Non Converters-MCI		Converters-MCI		
**SOA 250 ms**										
	**Mean**	**SD**	**Mean**	**SD**	***P***	**Mean**	**SD**	**Mean**	**SD**	***p***
**Words**	813.37	248.266	1046.15	375.320	<0.001	1052.94	366.878	1036.24	387.545	n.s.
**No words**	1205.38	483.902	1573.38	471.991	<0.001	1547.61	480.800	1610.85	456.888	n.s.
***p***	<0.001		<0.001			<0.001		<0.001		
**SOA 900 ms**										
	**Mean**	**SD**	**Mean**	**SD**	***P***	**Mean**	**SD**	**Mean**	**SD**	***p***
**Words**	808.15	247.161	1002.23	331.279	<0.001	996.86	323.493	1010.02	342.552	n.s.
**No words**	1177.64	460.944	1543.75	462.437	<0.001	1519.25	468.131	1579.34	452.266	n.s.
***p***	<0.001		<0.001			<0.001		<0.001		

**Table 2:** Priming Effect at SOAs 250 ms and 900 ms.

	HS		MCI		No Converters-MCI		Converters-MCI	
**SOA 250 ms**								
	**Mean**	**SD**	**Mean**	**SD**	**Mean**	**SD**	**Mean**	**SD**
**Related words**	778.27	187.428	944.01	248.428	969.00	229.787	923.58	261.521
**Unrelated words**	814.46	212.969	985.38	253.232	1029.33	246.156	949.46	253.915
***p***	0.015		0.003		0.038		0.321	
**Cohen’s d**	0.18		0.17		**0.25**		0.10	
**SOA 900 ms**								
	**Mean**	**SD**	**Mean**	**SD**	**Mean**	**SD**	**Mean**	**SD**
**Related words**	776.68	214.317	922.60	234.317	930.11	220.048	913.64	250.716
**Unrelated words**	806.21	204.353	962.37	242.047	982.23	244.109	938.63	238.152
***p***	0.071		0.003		0.010		0.745	
**Cohen’s d**	0.07		0.17		**0.22**		0.10	

## Appendix A. List of Items

**TARGET WORD**	**RELATED PRIME**	**UNRELATED PRIME**
vinegar	Salad	Hawk
student	Schoolchildren	Peacock
aerial	Cable	Walnut
banana	Gorilla	Die
mouth	Tongue	Species
orange	Peel	Sign
Dog	Peace	Owner
song	Voice	Point
horse	Animal	Example
colour	Red	Need
deluge	Thunder	Ram
dragon	fairy tales	Lip
grass	Lawn	People
summer	Sun	Land
river	Water	Game
giraffe	Height	Accent
Trip	Coach	Gasp
Ant	Bug	Candle
winter	Snow	Number
lake	Fish	Time
snail	Shell	Wax
mum	Sun	News
Sea	Island	Year
pencil	Case	Diamond
mule	Stable	Witch
nose	Face	Day
Ship	Voat	Arm
grandchild	Family	Answer
gandfather	Father	Bottom
track	Trace	Fear
vegetable garden	Vegetable	Loft
package	Courier	Bundle
planet	Heart	Minute
Rain	Weather	Type
spider	Venom	Steam
noise	Sound	Begin
classroom	School	Cold
seed	Grain	Injury
sister	Brother	Space
sauce	Tomato	Sheet
nest	Hibernation	Paint
roof	House	Name
cough	Sickness	Can
trumpet	Brass	Mill
coach	Binary	Steak
wind	Air	Part
worm	Maggot	Elf
volcano	Eruption	Reflex
Paw	Claw	Door
mosquito	Sting	Chest
